# Molecular modelling of mitofusin 2 for a prediction for Charcot-Marie-Tooth 2A clinical severity

**DOI:** 10.1038/s41598-018-35133-9

**Published:** 2018-11-15

**Authors:** Małgorzata Beręsewicz, Łukasz Charzewski, Krystiana A. Krzyśko, Andrzej Kochański, Barbara Zabłocka

**Affiliations:** 1 Molecular Biology Unit, Mossakowski Medical Research Centre, PAS, 02-106 Warsaw, 5 Pawińskiego St, Poland; 2 Faculty of Physics, University of Warsaw, 02-093 Warsaw, 5 Pasteura St, Poland; 3 Neuromuscular Unit, Mossakowski Medical Research Centre, PAS, 02-106 Warsaw, 5 Pawińskiego St, Poland

## Abstract

Charcot-Marie-Tooth disease type 2A (CMT2A) is an autosomal dominant neuropathy caused by mutations in the mitofusin 2 gene (*MFN2*). More than 100 *MFN2* gene mutations have been reported so far, with majority located within the GTPase domain encoding region. These domain-specific mutations present wide range of symptoms with differences associated with distinct amino acid substitutions in the same position. Due to the lack of conclusive phenotype-genotype correlation the predictive value of genetic results remains still limited. We have explored whether changes in the protein structure caused by *MFN2* mutations can help to explain diseases phenotypes. Using a stable protein model, we evaluated the effect of 26 substitutions on the MFN2 structure and predicted the molecular consequences of such alterations. The observed changes were correlated with clinical features associated with a given mutation. Of all tested mutations positive correlation of molecular modelling with the clinical features reached 73%. Our analysis revealed that molecular modelling of mitofusin 2 mutations is a powerful tool, which predicts associated pathogenic impacts and that these correlate with clinical outcomes. This approach may aid an early diagnosis and prediction of symptoms severity in CMT2A patients.

## Introduction

Charcot-Marie-Tooth disease type 2A (CMT2A) is an autosomal dominant axonal peripheral neuropathy caused by mutations in the mitofusin 2 gene (*MFN2*) [MIM: 608507]. The disease presents complex phenotypes including not only neuropathy-related features but also impairment of the central nervous system, sensorineural hearing loss, optic atrophy and these vary with severity and time of onset^1–3^. In genetic counseling, a reliable pathogenicity assessment of *MFN2* mutations is still needed.

Mitofusin 2 is a GTPase protein present in the outer mitochondrial membrane and responsible for regulation of mitochondrial network architecture via the fusion of mitochondria and in endoplasmic reticulum-mitochondria juxtaposition^4–7^. Fusion is an essential process for maintaining cellular dynamics because it allows exchange of contents, mtDNA and metabolites between neighboring mitochondria. MFN2, together with mitofusin 1, forms homo- and hetero-oligomers that promote tethering of adjacent mitochondria outer membranes and mediate their fusion^8,9^. Membrane fusion is a GTP-dependent process that depends on mitofusin 2 GTPase activity and consists of the following sequence of events: (i) binding of GTP to the GTPase domain leading to conformational changes of the GTP binding site; (ii) dimerization of two adjacent MFN2 molecules, which facilitates membrane proximity and their tethering and (iii) GTP-dependent outer membrane fusion associated with proper closure of mitofusin 2^10^. Several studies have demonstrated that *MFN2* mutations affecting its GTPase domain (95–339 aa) alter mitochondria fusion, leading to changes in the mitochondrial shape from tubular to more oval^9,11^. Moreover, in mitofusin 2-mutant cells, mitochondria seem to extensively aggregate around nucleus and are nonfunctional for fusion^9,12^. Mitochondrial network dysfunctions were also accompanied by reticular stress, diminished respiratory capacity and changes in mtDNA in patient–derived fibroblasts^11–13^. Another report suggested that mitochondrial network dysfunction is associated with impaired GTPase activity^9^ while we have recently reported that p.Arg274Trp mutation does not influence GTP binding or GTPase activity^11^. All these indicate significant functional heterogeneity of *MFN2* mutants that underlie CMT2A.

So far, more than 100 *MFN2* gene mutations have been reported of which majority is located within the GTPase domain-encoding region. Patients carrying mutations within GTPase domain present wide range of symptoms starting from classical form of CMT2A and ending with impairment of the central nervous system^2^. Moreover, significant differences in symptoms are also noticeable when distinct amino acid substitutions occurs at the same position^11,14^. Therefore, given that it is difficult to establish a categorical genotype - phenotype correlation, we have investigated whether the protein structure alterations caused by mutations in the *MFN2* gene can inform the diseases phenotypes.

We collected information on all the GTPase domain mutations reported in the literature. Of these, 26 mutations resulting in an amino acid in a position being replaced by no fewer than two distinct amino acids were selected for further analysis. Using a stable model of MFN2, described earlier^11^, we evaluated the effects of each mutation on the protein structure and, on this basis, predicted the molecular/functional consequences. It turned out that mutation effects are related to various stages of the fusion process, such as disturbances in the rearrangement of the GTP binding site, impairment of mitofusin 2 dimerization, of GTP hydrolysis and also MFN2 closure, which is an essential element in the fusion process^10^. In parallel, the clinical features associated with the given mutation and described in the literature, were carefully analyzed in an unbiased manner and our own internal score of neurological deficits has been generated. Finally, both analyzes were cross-referenced to establish whether specific protein structure changes caused by the mutation position and properties of substituted amino acids correlate with the observed symptoms.

As a result, we established that using the newly obtained stable model of mitofusin 2 and the available molecular modelling tools it is possible to predict the pathogenic effect of a mutation in the *MFN2* gene and to anticipate the patient’s prospective clinical outcome.

## Results

Of all 68 identified mitofusin 2 mutations within the GTPase domain, 26 in which the amino acid in the same position was replaced by at least two other amino acids were selected for further analysis. Nominated mutations in 11 groups, together with corresponding clinical features of patients are listed in Table 1. We developed our own phenotypic assessment scale to determine symptom severity of each patient. Then, the potential impact of each mutation on the MFN2 structure was evaluated using molecular modelling. Finally, both analyses were combined to answer if predicted molecular effects caused by a specific mutation correlate with the phenotype severity.

**Table 1:** Clinical and electrophysiological characteristics of CMT2A patients bearing mutation within *MFN2* GTPase domain.

Nucleotide change	Protein change	Clinical details			Phenotype		Score	References
		Age of onset (years)	CMAP (median nerve) [mV]	CMTNS/FDS	Peripheral neuropathy	CNS/cranial nerves		
c.311 G > A	p.Arg104Gln	—	—	−/−	healthy individual with normal results of electrophysiological examination	—	0	^27^
c.310 C > T	p.Arg104Trp	1–10	0.0, 0.1, 0.8, 1.6; 1.8, 2,7	22/7^26^	early onset CMT2A (1.5) *or* classical CMT2A (1)	pyramidal signs (2) optic atrophy (1) mental retardation (1) other minor symptoms (0.5)	5.5–6	^15, 25, 26, 28– 30^
c.380 G > A	p.Gly127Asp	16	11.9	4/2	classical CMT2A (1)	extensor plantar responses (2)	3	^2^
c.380 G > T	p.Gly127Val	6–62	0.8, 2.0, 8.4	−/−	late onset CMT2A (0.5) *or* classical CMT2A (1)	—	0.5–1	^35^
c.494 A > G	p.His165Arg	6–16	1.0, 5.2, 11.5, 20.1	5/1	classical CMT2A (1)	subcortical lesions in MRI (0.5) sensorineural hearing loss (0.5)	2	^2, 3, 22^
c.493 C > G	p.His165Asp	4–20	5.0, 5.0, 7.0, 11.0	−/−	classical CMT2A (1) *or* early onset CMT2A (1.5)	pyramidal signs (2)	3–3.5	^21, 24^
c.494 A > T	p.His165Leu + ALS	14	2.2	−/−	classical CMT2A (1) ALS is rather casual and not associated with *MFN2* mutation	—	1	^23^
c.493 C > T	p.His165Tyr	12	—	−/−	classical CMT2A (1)	—	1	^3^
c.629 A > T	p.Asp210Val	1.5	—	−/−	early onset CMT2A (1.5)	pyramidal signs (2) cerebellar ataxia (0.5) deafness (0.5) optic atrophy (1) cataracts (0.5) learning difficulties (0.5) mitochondrial myopathy (0.5)	7	^12^
c.628 G > T	p.Asp210Tyr	0.5	no values	−/−	early onset CMT2A (1.5)	pyramidal signs (2) microcephaly (0.5) tremor (0.5) sensorineural hearing loss (0.5) optic atrophy (1) developmental delay (1)	7	^36^
c.730 G > C	p.Val244Leu	4	6.3	−/−	early onset CMT2A (1.5)	periventricular leukomalacia (0.5)	2	^40^
c.730 G > A	p.Val244Met	<5	—	−/−	early onset CMT2A (1.5)	—	1.5	^15, 39^
c.749 G > A	p.Arg250Gln	12–21	—	−/−	late onset CMT2A (0.5) *or* classical CMT2A (1)	—	0.5–1	^3, 37^
c.748 C > T	p.Arg250Trp + Arg400* + Arg476*	4–10	2.7	−/−	classical CMT2A (1) *or* early onset CMT2A (1.5)	—	1–1.5	^3, 38^
c.751 C > G	p.Pro251Ala	8–50	—	−/−	classical CMT2A (1) tremor (0.5)	—	1.5	^31^
c.752 C > G	p.Pro251Arg	1, 2	—	11–20/−	early onset CMT2A (1.5) wheelchair (1)	—	2.5	^37, 41^
c.752 C > T	p.Pro251Leu	25	—	13/2	late onset CMT2A (0.5)	—	0.5	^42^
c.775 C > T	p.Arg259Cys	>30	decreased	−/−	late onset (0.5)	sudden visual loss (1.5)	2	^17^
c.776 G > T	p.Arg259Leu	19	—	−/−	classical CMT2A (1)	mild pyramidal signs (1.5)	2.5	^18^
c.776 G > C	p.Arg259His	17	—	10/2	classical CMT2A (1)	—	1	^19^
c.821 G > A	p.Arg274Gln	11–35	—	−/−	classical CMT2A (1)	—	1	^31^
c.820 C > T	p.Arg274Trp	10	—	−/−	classical CMT2A (1)	markedly reduced nerve conduction velocity in the motor fibers of the median nerve (0.5) proximal weakness (0.5) mental retardation (1)	3	^1^
c.827 A > G	p.Gln276Arg	10	—	−/−	classical CMT2A (1)	optic nerve atrophy (1)	2	^32^
c.828 G > C	p.Gln276His	9	decreased	−/−	classical CMT2A (1)	optic nerve atrophy (1)	2	^33^
c.830 A > G	p.His277Arg	<15	—	−/−	classical CMT2A (1)	—	1	^3^
c.829 C > T	p.His277Tyr	<10	—	−/−	classical CMT2A (1)	pyramidal signs (2) vasomotor troubles (0.5)	3.5	^15^

Legend: “-“ the results of the analysis were not reported, “−/−“ nor CMTNS, neither FDS score was reported, CMTNS-Charcot Marie Tooth Neuropathy scale, FDS- Functional disability scale, CMAP- compound muscle amplitude potential, CNS-central nervous system.

First, based on our analysis it turned out that mutation effects are related to various stages of the fusion process, such as (i) disturbance in the rearrangement of the GTP binding site (His277), (ii) impairment of MFN2 dimerization (Arg104, His165, Arg259, Arg274, Gln276), (iii) impairment of mitofusin 2 GTP hydrolysis (Gly127), (iv) impairment in MFN2 closure, which is an essential element in the fusion process (Asp210, Arg250) and (v) other yet unknown mechanisms (Val244, Pro251).

### Disturbance in rearrangement of the GTP binding site

Binding of GTP to the GTPase domain leads to conformational changes of the GTP binding site, which is an essential element of stabilization of the attached ligand and promotion of subsequent dimerization. Histidine in the position 277 is an amino acid significantly involved in this process. Binding of GTP initiates sequential intramolecular interactions between His277 and its adjacent amino acids (Asn257, Cys281, Ser229 for details see Supplement, Fig. S1) which guarantees correct rearrangements of GTP binding site. Two different substitutions in His277 have been reported in CMT2A patients, to date (Table 1). The p.His277Arg mutation is responsible for the classical phenotype and assigned 1 point in our phenotypic scale^3^. The p.His277Tyr mutation is associated with a more severe phenotype where classical neuropathy is associated with pyramidal signs and vasomotor abnormalities^15^. According to our phenotypic assessment scale, this mutation receives 3.5 points. Molecular dynamics simulations revealed that both mutations lead to the disruption in intramolecular interactions leading to impaired rearrangement of the mitofusin 2 GTP binding site (Supplement, Fig. S1). However, comparison of molecular consequences of these two mutations is ambiguous, both retain certain parts of wild type interactions and behavior. In the current state of knowledge it is unclear which of them might generate stronger disturbances in MFN2 mechanistic properties, while clinical studies reveal that the p.His277Tyr mutation is responsible for more severe phenotype. Most likely, His277 is involved in another unknown aspect, therefore further studies are needed.

### Impaired dimerization of mitofusin 2

Arg104, His165, Arg259, Arg274 and Gln276 seem to be engaged in the dimerization process of two adjacent mitofusin 2 molecules. The involvement of these amino acids may be direct, as in the case of Arg259 and His165, or indirect as in the case of Arg104, Arg274 and Gln276. Dimerization facilitates membrane proximity, tethering and fusion of mitochondrial outer membranes.

Arg259 is the key amino acid responsible for the dimerization of mitofusin 2. It is known that mutagenesis of analogical residue in mitofusin 1 (p.Arg238Ala) prevents its dimerization^16^. We have found three different substitutions in Arg259: p.Arg259Cys, p.Arg259His and p.Arg259Leu^17–19^. The p.Arg259His mutation is responsible for the classical phenotype and assigned 1 point in our phenotypic scale^19^. Patient bearing the p.Arg259Cys mutation presents late onset disease and severe sudden visual loss^17^. According to our assessment scale, this was rated at 2 points. In contrast, the p.Arg259Leu variant has been associated with a classical CMT2A phenotype with accompanying pyramidal signs, which was rated at 2.5 points^18^. Molecular dynamics simulations indicate that leucine and cysteine substitutions have similar deleterious effects on mitofusin 2 structure (Supplement, page 2). Both prevent dimerization and probably also disrupt the hydrolysis of GTP. Moreover, it seems that the effect of these two mutations can be more deleterious than the effect caused by histidine substitution. Hence, it is not surprising that patients with p.Arg259Cys and p.Arg259Leu mutations manifest with CMT2A with additional symptoms such as the involvement of the central nervous system, albeit affecting different regions (the optic nerve and pyramidal tracts). The above comparison reveals that predicted changes in the mitofusin 2 structure correlate with the observed symptoms (Table 2).

**Table 2:** Comparison of clinical outcome and the predicted changes in the mitofusin 2 structure due to mutations position and properties of substituted amino acids.

Amino acid position	Clinical outcome	Structure impairment
Arg104	Trp > Gln	Trp > Gln
Gly127	Asp > Val	Asp > Val
His165	Asp > Arg > Leu = Tyr	Asp > Arg > Leu = Tyr
Asp210	Tyr = Val	Tyr > Val
Val244	Leu $\ge$ Met	Leu = Met
Arg250	Trp > Gln	Trp > Gln
Pro251	Arg > Ala > Leu	Arg > Ala = Leu
Arg259	Leu $\ge$ Cys > His	Leu = Cys > His
Arg274	Trp > Gln	Trp > Gln
Gln276	Arg = His	Arg = His
His277	Tyr > Arg	Tyr = Arg

Another residue, which mutation leads to dimerization impairment is His165. Analogously to mitofusin 1, upon GTP hydrolysis, tightening of two adjacent mitofusins 2 occurs resulting in formation of new contacts between its subunits^20^. The following amino acids are responsible for these new intermolecular interactions: His165-Glu268 and His168-Glu272 (Supplement, Fig. S2) and Lys120-Glu266.

His165 can be substituted by four other amino acids: arginine, aspartic acid, leucine and tyrosine, which all produce CMT2A phenotype^2,3,21–24^. Patients with p.His165Leu and p.His165Tyr mutations present a classical CMT2A and therefore, these were assigned 1 point^3,23^. It is important to note that in the patient with the p.His165Leu mutation the classical CMT2A was accompanied by motor neuron disease, consistent with amyotrophic lateral sclerosis (ALS)^23^. Most likely, the co-occurrence of these two rare neuromuscular disorders was a coincidence. Patients bearing p.His165Arg mutation, in addition to typical neuropathy, developed other symptoms such as subcortical lesion and sensorineural hearing loss^2,3,22^. In the patient harboring p.His165Asp mutation CMT2A with pyramidal signs were diagnosed^21,24^. Therefore, we assigned the patient with p.His165Arg mutation 2 points and the patient with p.His165Asp 3–3.5 points in our internal score. Substitutions of histidine to leucine or tyrosine at position 165 do not cause significant changes in the mitofusin 2 structure which correlates with the classical CMT2A phenotype observed in patients with these mutations. Both mutations disables intermolecular interactions with Glu268 and any other effects are not observed. In contrast, substitution to arginine or aspartic acid causes minor or major disturbances in interactions with Glu268, respectively and this is reflected in various phenotypes, all more severe than classical CMT2A (Supplement, Fig. S2). Most probably the observed phenotypes are a consequence of disordered mitofusin 2 dimerization. p.His165Asp mutation leads to unfavorable contact with Glu268 preventing dimer formation, while p.His165Arg strengthens the interaction with Glu268, potentially impeding mitofusin 2 dissociation.

Concluding, in accordance with bioinformatics analysis, the clinical outcome of Tyr and Leu substitutions at the 165^th^ position are limited to the classical phenotype, while arginine or aspartic acid substitutions reflect in various phenotypes, all more severe than classical CMT2A. It is another example where predicted changes in the mitofusin 2 structure are mirrored in clinical outcome.

**Figure 1:**
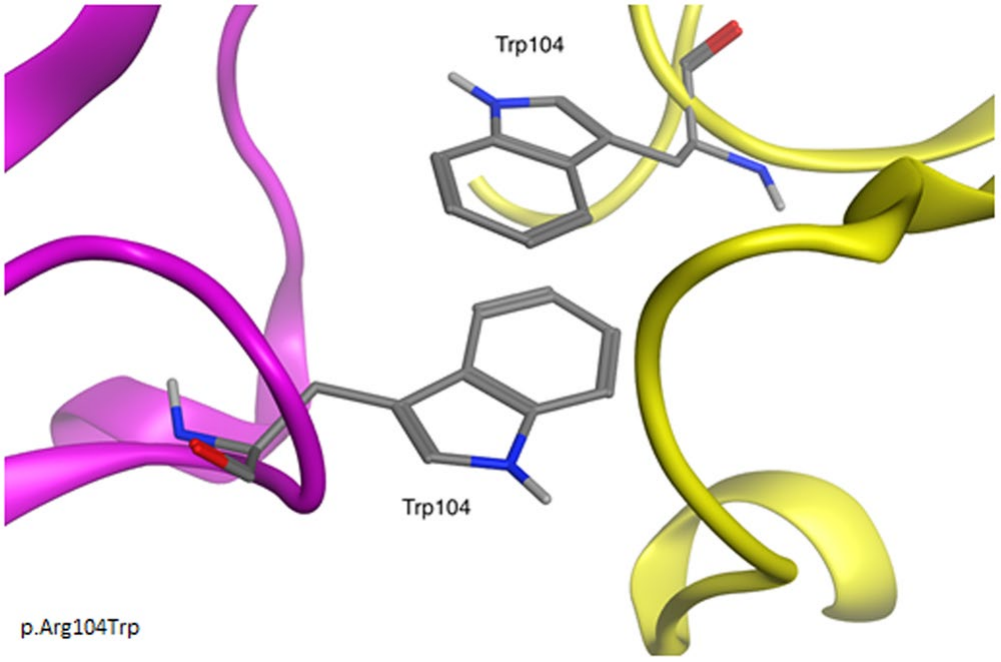
Trp104 of the two opposite subunits of mitofusin 2 form contact with each other through the stacking interaction. Such interaction is absent in WT protein.

Two mutations at Arg104 position: p.Arg104Gln and p.Arg104Trp have been found^15,25–30^. The p.Arg104Trp mutation was identified by a number of independent groups^15,25,26,28–30^. All reports agreed that this variant is responsible for an extremely severe phenotype, where the classical CMT2A is accompanied by symptoms such as pyramidal signs, optic atrophy and mental retardation. The described symptoms however, were slightly different between case reports hence these patients were assigned by us from 5.5 to 6 points. Molecular dynamics revealed that in the dimer structure where both subunits carry this mutation, the side chains of Trp104 form a stacking interaction that stabilizes the dimer and hinders its decomposition (Fig. 1).

The p.Arg104Gln amino acid substitution was identified in a healthy carrier, in whom electrophysiological examination did not reveal any abnormality^27^. Thus, in terms of autosomal dominant trait of inheritance, this *MFN2* gene variant may be classified as harmless polymorphism or extremely weak mutation acting in autosomal recessive manner. Similarly, in molecular modelling experiments, this substitution seems not to influence the dimer stability. This indicates that the substitution to glutamine at position 104 does not affect the mitofusin 2 structure while the substitution to tryptophan has deleterious effect (Supplement, page 4). This result is consistent with the presence of two completely different phenotypes and confirms that molecular modelling reflects clinical outcomes of CMT2A.

Arg274 is an amino acid that indirectly participates in mitofusin 2 dimerization since it is involved in the formation of GTP binding site. Two different substitutions of arginine at position 274 such as p.Arg274Gln and p.Arg274Trp have been identified^1,31^. The patient bearing the p.Arg274Trp mutation presents with a moderate, early-onset CMT2A with peripheral axonal neuropathy coexisting with mental retardation^11^ while clinical course of the p.Arg274Gln was clearly milder. According to our assessment scale the patient with p.Arg274Gln mutation received 1, while the p.Arg274Trp 3 points.

In our simulations of both mutations, we observed a mechanics disorder that stabilizes the GTPase domain in the inactive state, resulting in dimer formation regardless of the GTP binding (Supplement, Fig. S3). It seems also that the p.Arg274Trp substitution has more deleterious effect on mitofusin 2 structure than p.Arg274Gln what correlates with more severe phenotype.

Another amino acid involved in mitofusin 2 dimerization is Gln276 located close to the Glu272, which similar to His165-Glu268 is responsible for intermolecular interactions in dimeric form.

Two substitutions of Gln276: p.Gln276Arg and p.Gln276His have been described^32,33^. Patients, regardless of the mutation, present classical CMT2A with accompanying optic nerve atrophy - each was assigned 2 points. Molecular dynamics simulations indicate that both mutations have similar effect on mitofusin 2 structure and promote the formation of new interactions with Gly272 within a single molecule which can affect the dimer formation (Supplement, Fig. S4). Due to the presence of the classical CMT2A phenotype and optic nerve atrophy in the patients with both mutations, we suggest that these variants induce the same molecular defect within MFN2 protein.

### Impaired GTP hydrolysis

For GTP hydrolysis the following processes must take place: (i) binding of GTP to the GTPase domain and (ii) dimerization of two adjacent MFN2 molecules including transient dimerization of the GTPase domains in a parallel head-to-head fashion^34^.

His128 is an amino acid significantly involved in this process and assisted by Gly127 which maintains the water molecule in the proper position during the GTP hydrolysis (Supplement, Fig. S5). Two different substitutions have been identified in 127 position so far, including the p.Gly127Val and p.Gly127Asp^2,35^. The resulting clinical characteristics were significantly different – from patient with mild phenotype (p.Gly127Val) to the patient who, in addition to the classical CMT2A, had also pyramidal signs (p.Gly127Asp) indicating involvement of the central nervous system. According to our scale, the p.Gly127Val mutation was assigned 0.5–1 while p.Gly127Asp was given 3 points. Molecular dynamics simulations indicate that p.Gly127Val mutation impairs His128 placement for GTP hydrolysis, while the p.Gly127Asp mutation disturbs not only GTP hydrolysis but also mitofusin 2 dimerization. It indicates that p.Gly127Asp variant has more deleterious effect on MFN2 structure which correlates with more severe phenotype in comparison to p.Gly127Val.

### Impaired MFN2 closure

**Figure 2:**
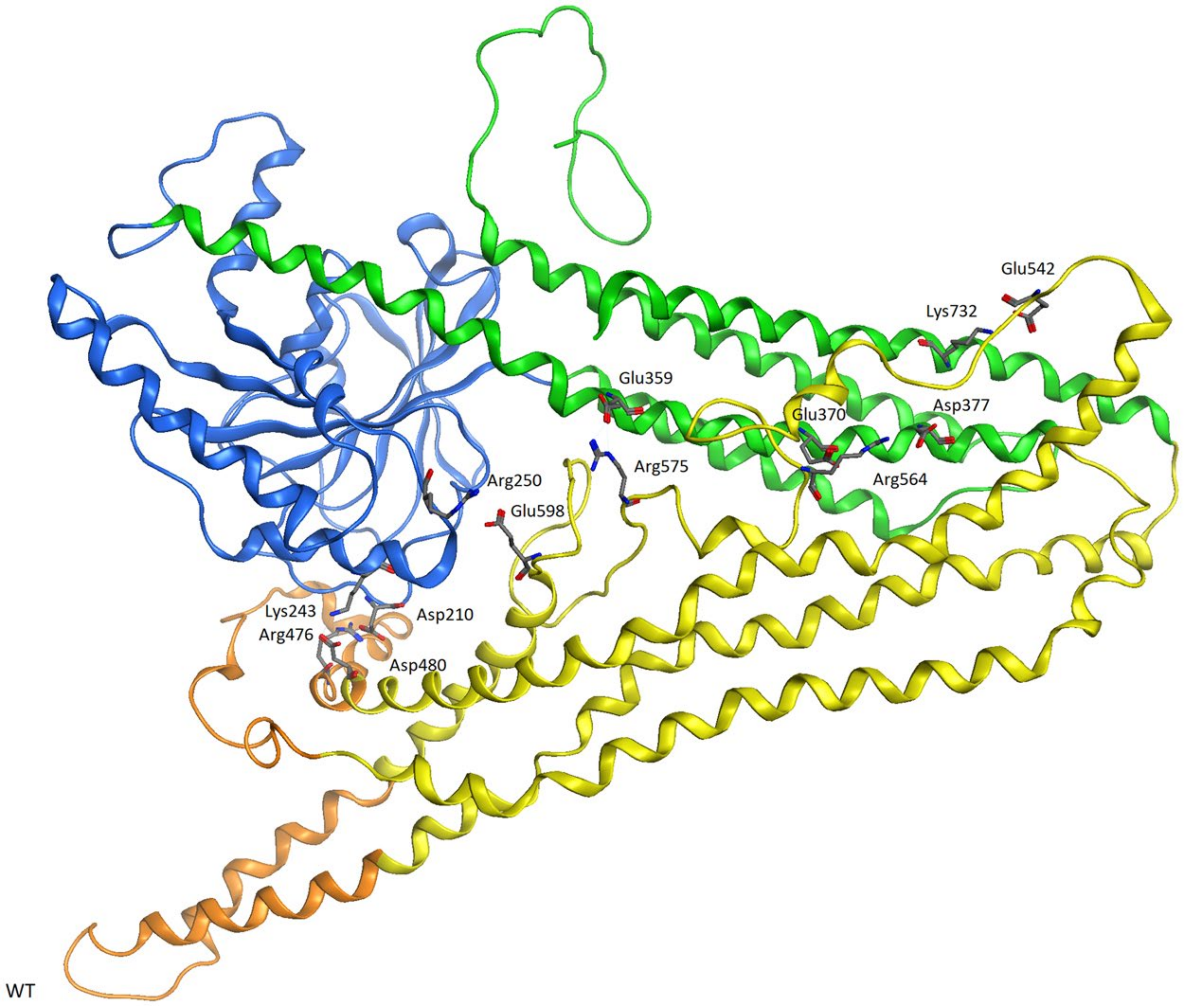
The following amino acids (sticks) form electrostatic intramolecular interactions driving MFN2 closure: Asp210 (GTPase) - Arg476 (HR2) - interaction tightening paddle domain, Lys243 (GTPase) - Asp480 (HR2) - interaction tightening paddle domain, Arg250 (GTPase) - Glu598 (HR2) - interaction tightening HR2 domain, Glu542 (HR2) - Lys732 (HR1) - interaction tightening HR2 domain, Asp377 and Glu370 (HR1) - Arg564 (HR2) - interaction tightening HR1 domain, Glu359 (HR1) - Arg575 (HR2) - interaction tightening HR1 domain. Colors represent domain composition: GTPase (blue), HR1 (green), HR2 (yellow) and paddles (orange).

In order to conduct membrane fusion, mitofusins undergo domain reorientation rendering closed, diamond-shaped structure. We have identified internal long-range factors (electrostatic interactions) within individual domains enabling such closure (Fig. 2).

Around these specific amino acids there are residues, which “tighten” the structure at a smaller distance by Van der Waals and hydrogen bonds interactions. Therefore, mutations that destroy the above electrostatic interactions cause difficulties in the mitofusin 2 closure. The amino acids initiating and terminating the closing of the structure are the most important. In this study, among the mutations selected for analyses, four affect amino acids essential for proper MFN2 closure (Asp210 and Arg250). Two mutations at the codon 210 involving substitutions to valine and tyrosine result in an extremely severe mitochondrial, systemic disorder phenotype associated with axonal neuropathy^12,36^. Regardless of the mutation, patients presented with an early-onset disease with pyramidal signs and optic atrophy. Therefore, both p.Asp210Val and p.Asp210Tyr mutations were equally rated at 7 points. Molecular dynamics simulations reveled that both variants impair mitofusin 2 closure with the difference, that substitutions to tyrosine seems to be more deleterious than valine (Supplement, page 7). This is not entirely consistent with the clinical data of patients which were equally evaluated by us.

Another group of mutations affects arginine at position 250 that, similarly to Asp210, is engaged in creation of the interdomain interaction. Arginine substitutions by glutamine and tryptophan (p.Arg250Gln, p.Arg250Trp) were described^3,37,38^. The glutamine substitution was reported by two independent groups^3,37^. Both cases presented mild CMT2A and differed only in symptom onset times. Patient with the later onset, described by Verhoeven and coworkers was rated at 0.5 while, one described by McCorquodale and coworkers diagnosed at the age of 12, was rated at 1 point. The p.Arg250Trp substitution was associated with an additional inherited in *trans* stop codon change of arginine at positions 400 or 476^3,38^ (nonsense mutations are predicted to behave as a not disease causative). The p.Arg250Trp mutation causes a mild neuropathy with early age of onset, thus rated at 1.5 points.

Molecular dynamics simulations reveled that both variants impair mitofusin 2 closure with the difference, that substitutions to tryptophan seems to be more deleterious than glutamine (Supplement, page 8). Therefore, of these two mutations, the p.Arg250Trp should cause a more severe phenotype. This molecular prediction is consistent with the clinical features.

Amino acids at positions 210 and 250 are involved in mitofusin 2 closure, which is an essential step in the mitochondrial fusion. Interestingly, mutations at these positions produce very distinct phenotypes with a more severe one for position 210. Most likely, this amino acid plays a much more important role in the mitofusin 2-closure hierarchy than the amino acid at 250 position. Thus, mutation in 210 amino acid is associated with more severe symptoms.

### Unknown mechanisms

**Figure 3:**
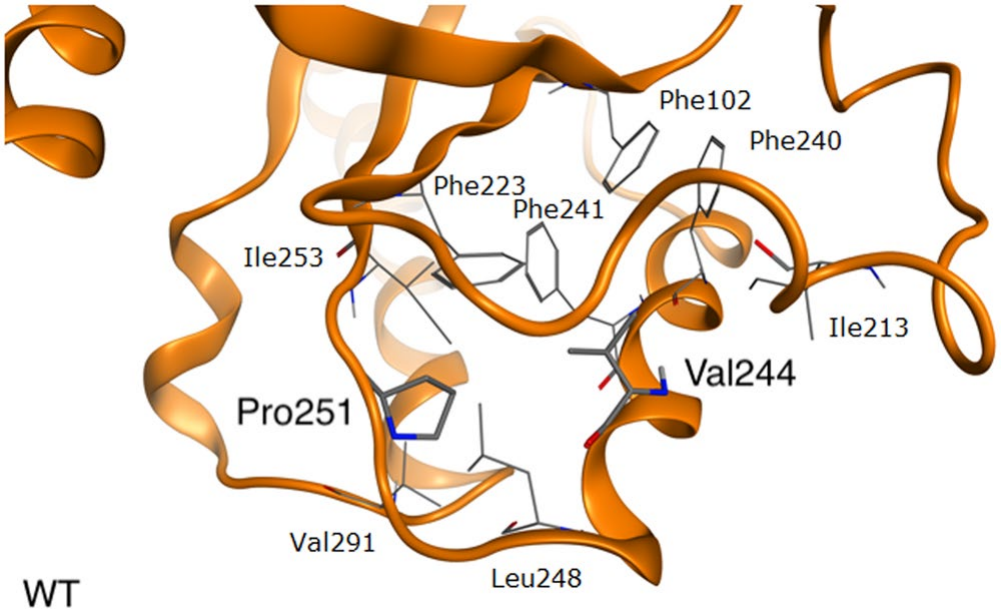
In wild type mitofusin 2 Pro251 and Val244 sidechains are oriented towards the hydrophobic interior of the protein forming Van der Waals interactions. Reported mutations in both positions (p.Val244Leu, p.Val244Met, p.Pro251Ala, p.Pro251Arg, p.Pro51Leu) do not cause significant structural changes.

Two more mutations affecting Val244 (p.Val244Leu and p.Val244Met) have been identified^15,39,40^. Leucine and methionine, like valine, are hydrophobic amino acids, although they are longer. Their side chains are located in this hydrophobic region and don’t disturb it - in simulations we do not observe any significant structural changes. At this moment it is difficult to explain in what processes they might be involved (Fig. 3). Nevertheless, patients with these mutations present with an early onset CMT2A and the p.Val244Leu variant was additionally linked to periventricular leukomalacia^40^. Therefore, p.Val244Met and p.Val244Leu mutations were assigned 1.5 and 2.0 points, respectively^15,39^.

In case of Pro251 three mutations have been identified, namely p.Pro251Ala, p.Pro251Arg and p.Pro251Leu^31,37,41,42^. The most severe phenotype of the wheel-chair dependency resulted from the p.Pro251Arg mutation^37,41^. In the patient harboring the p.Pro251Ala mutation tremor accompanied the classic CMT2A phenotype while the p.Pro251Leu mutation resulted in a classical CMT2A with late age of onset^31,42^. Therefore, p.Pro251Arg, p.Pro251Ala and p.Pro251Leu mutations were assigned 2.5, 1.5 and 0.5 points, respectively.

At the molecular level, leucine and alanine share the same chemical characteristics as proline (Fig. 3). While, leucine is slightly larger, alanine is smaller than proline and for these no significant changes have been observed in simulations. Arginine is a much larger amino acid and also charged, therefore it does not match the hydrophobic environment of that region. It’s side chain comes out and attempts to interact with Glu598 (HR2), but the distance between these residues is too large to form a strong contact. While the Arg251 side chain exits that region, it does not destroy the mitofusin 2 backbone structure.

Based on the chemical properties of these substitutions, it could be predicted that arginine should have a much more deleterious effect on structure than leucine or alanine. In full agreement with this prediction, the patient with p.Pro251Arg presented with a more severe phenotype. However, we are currently not able to explain the phenotypic differences between p.Pro251Ala, and p.Pro251Leu variants.

In summary, of 11 tested groups of analyzed mutations (Table 1) positive correlation of molecular modelling and the clinical features has been demonstrated for 8, which constitutes 73% of agreement (Table 2).

### Prediction of clinical outcome severity by mutation assessment tools

To compare the results of our analysis to those derived from existing predictors of clinical significance, the following mutation assessment tools were used: PROVEAN, Fathmm, MutationTaster, PolyPhen-2 and Mutation Assessor. All used tools cluster analyzed mutation as deleterious with one exception - the PROVEAN has classified p.Arg250Gln as neutral. The obtained predictions allowed us to organized the analyzed mutation according to their potential severity (Supplement, Table S1), thus in turn was correlated with clinical features associated with a given mutation. Depending on the test, we received 18–45% compatibility between the clinical features and mutation assessment. The highest correlation was obtained for Mutation Assessor, PROVEAN and Fathmm.

## Discussion

Here, we present a comprehensive bioinformatics analysis of the molecular effects of selected *MFN2* mutations affecting the GTPase domain, which were correlated with the clinical features of patients bearing these mutations. Eight groups out of eleven of analyzed mutations showed a positive correlation of molecular modelling with the clinical features indicating 73% agreement (Table 2). In three groups (p.Asp210, p.Pro251, p.His277) compatibility between the comparative protein modelling and the severity of clinical symptoms was incomplete but not discordant. Therefore, the study of the effects of mutations by comparative molecular modelling methods might be helpful in predicting and explaining the pathogenicity of CMT2A, irrespective of incomplete knowledge about the dynamics of mitofusin 2.

Since the patients analyzed here were examined by different neurologists using various examination schemes we had to abandon the use of the standard Charcot-Marie-Tooth disease (CMT) neuropathy score (CMTNS)^43^ in favor of our own scoring system for the clinical evaluation.

We are aware that creating a uniform scale based on the literature data can limit the validity of our results e.g. we cannot exclude other causes, not described in the publications and/or identified later that might be responsible for the development of non-classical CMT2A symptoms. Noteworthy, in case of CMT2A patients manifesting with additional symptoms and, in some cases, even with systemic mitochondrial disorder, the usage of CMTNS focused on the severity of peripheral neuropathy does not reflect a complete phenotype. In the creating of our assessment scale we decided to treat CMT2A disease as a systemic mitochondrial disorder. Therefore, every additional symptom of mitochondrial disease (myopathy, hearing impairment, visual loss, demyelinating changes in the central nervous system, etc.) was taken into account in our scale. Parallel to CMNTS, the Functional Disability Scale (FDS) is sometimes used. FDS reflects severity of the motor deficit from normal subjects (0 points) to the bedridden patients (8 points). However, even in coexistence with CMTNS, FDS does not reflect systemic mitochondrial damage observed in some CMT2A patients. Moreover, in mutation analyzed by us information about CMNTS and FDS are available only for limited group of patients. Thus, we were not able to compare the clinical severity using both CMTNS and FDS.

In contrast, our score reflects the sequence of processes occurring in pathophysiology of mitochondrial disease evolving from peripheral nerve damage (one system) to the multisystem disease characterized by the involvement of the central nervous system and cranial nerves. In our opinion, this method of scoring could be serviceable for all CMT2A patients with mutations in the GTPase domain. Here, using a blind approach, we have reached a high level of compliance between the clinical picture of CMT2A and the corresponding structural analysis. This, on the one hand, confirms the effectiveness of our internal scoring method and, on the other hand, indicates the value of molecular modelling in the diagnosis process. Similar approach to understand the variability in the severity of CMT2A disease was proposed by Rouzier and coworkers^14^, and this will be discussed later.

Results obtained in molecular modelling showed 73% compatibility with the clinical outcome. While, the same comparison using commonly used mutation assessment tools (Supplement, Table S1) showed at most 45% compatibility indicating greater efficiency of our approach. The mutation assessments tools provide prediction regardless of the mutation localization. In our approach we compared the severity of mutations only in cases when variants are in the same amino acids position and affect the same biological function. This limitation, however, has its significant advantages, including greater efficiency prediction. Such approach is consistent with the clinical picture, which proves its performance. Therefore, the molecular modelling approach seems applicable for analysis where only one mutation occurs in particular site. As the analysis is based on the changes taking place in the proper structure it would be always possible to compare mutated system to wild-type and predict the severity.

Until now, all studies on the pathogenicity of mitofusin 2 mutations have been related to impaired function of MFN2 protein and its consequences. Therefore, the alterations were studied mainly in the mitochondrial and endoplasmic reticulum morphology, mtDNA integrity and respiratory chain activity in patient-derived cells and tissues. The problem with these studies, however, is that they are long-lasting and the access to the material is increasingly limited. Effects of p.Asp210Tyr mutation in muscle and skin biopsies were studied by Renaldo and coworkers^36^. The biopsy (*m. quadriceps*) showed denervation and revealed that most fibers were cytochrome c-oxidase negative. In muscle samples they showed decrease of mitochondrial complex I and III activities. Analysis of mitochondrial DNA from the muscle biopsy display significant reduction in the mitochondrial copy number. Moreover, in patient-derived fibroblasts, low global mitochondrial activity and poor cell respiration were observed. According to our structural analysis, Asp210 is considered as one of the amino acids responsible for forming interdomain interaction driving MFN2 structure closure, which is an essential element in the mitochondrial membrane fusion. Substitution of Asp210 by tyrosine prevents the interaction with Arg476 as well as Lys243-Asp480 (Fig. 2), which makes the enclosement of MFN2 difficult or even impossible. It is no wonder that a patient harboring p.Asp210Tyr mutations was evaluated by us at 7 points - as one of the most severe clinical case. Our comprehensive analysis of clinical data and the influence of mutation on molecular structure of MFN2 are consistent in terms of severity of symptoms and, additionally, they bring us closer to understanding the mechanism of protein function. Mutation in the same codon was reported by Rouzier and coworkers (p.Asp210Val) with the clinical course similar to p.Asp210Tyr^14^. Likewise to our study, authors compared impact of these two mutations affecting amino acid in position 210 in human mitofusin 2 structure^14^. The difference, however, is that our study was made on the whole structure of MFN2, while the Rouzier’s only on a fragment of protein covering residues from 122–306^14^. They found that Tyr in position 210 has more deleterious effect than Asp210Val mutation and this is in line with both their clinical assessment of these two patients and our structural analysis. Indeed, according to our data, Asp210Val substitution impairs the formation of intramolecular interactions but to a lesser extent than Asp210Tyr, even the studies present by Rouzier and coworkers are not entirely consistent with our assessment scale^14^. This may be caused by the obvious discrepancies between scoring method used by us and clinical assessment of neuropathy based on the direct observation and description of patients.

Another example is the case of p.Arg274Trp mutation described by us elsewhere^11^. We showed, using the patient-derived fibroblasts, that mitochondrial and endoplasmic reticulum morphology and mtDNA content were affected significantly by the presence of the mutant MFN2 protein. Subsequently, we revealed that the mutation did not affect the GTPase activity nor the GTP binding. Therefore, we suggested that the biological malfunctions observed in our *in vitro* experiments were not the consequences of impaired GTPase activity but rather reflected an impaired contribution from the GTPase domain into other MFN2 molecular activities involving that region. Here, employing molecular modelling we showed that p.Arg274Trp substitution leads to impairment of dimerization of two adjacent mitofusin 2 molecules. Moreover, dimerization impairment can cause extensive aggregation of nonfunctional mitochondria around nucleus what is actually visible in patient-derived fibroblasts^7^. The observed disturbances in protein structure correlate with the severity of symptoms in the patient. In molecular dynamics simulations of the p.Arg274Gln mutation we observed a much smaller effect than in p.Arg274Trp, which is consistent with the milder clinical phenotype described by Zuchner and coworkers^31^. This is also in line with data by Detmer and coworkers who showed that p.Arg274Gln mutation did not affect mitochondrial network in wild type MEFs and showed a considerable restoration of mitochondrial tubules in double Mfn-null cells^9^. Moreover, Detmer and coworkers assessed the effect of p.Pro251Ala on mitochondria morphology and ability to mediate mitochondrial fusion. In wild-type MEFs p.Pro251Ala caused substantial mitochondrial aggregation only when cells were transfected at high multiplicity of infection^9^. In our study, substitution of the Pro251 by alanine or leucine did not have a great impact on protein structure while substitution for arginine resulted in the appearance of a second positive charge on the protein surface (adjacent to Arg250). It might enhance the local charge in a region involved in domain rearrangement, which might stabilize the closed MFN2 conformer. Even though it was not clearly confirmed by molecular dynamics, it would justify the higher severity of disease symptoms.

In conclusion, we found here that molecular modelling of mitofusin 2 mutations is a powerful tool, which predicts associated pathogenic impacts and that these correlate with clinical outcomes. This approach may aid an early diagnosis and prediction of symptoms severity progression in CMT2A patients. To further confirm the prognostic value of bioinformatics analysis of MFN2 mutations, our results should be confirmed in a larger cohort of CMT2A patients. In terms of clinical studies, the group of CMT2A-affected patients described in the literature is still small. Nevertheless, our data indicates the predictive value of the MFN2 protein modelling, at least with respect to the mutations located within GTPase domain. There is a needed to study the specific correlation between MFN2 mutations affecting protein dynamics and cell defects in patients with complex and unusual phenotypes involving the central and peripheral nervous systems.

## Methods

### Analysis of *MFN2* gene mutations affecting its GTPase domain

According to our MFN2 structure prediction, the amino acids 95 to 339 form the GTPase domain. The following databases have been searched to collect all *MFN2* mutation affecting its GTPases domain: disease mutation database: Mitofusin 2 project (http://www.progettomitofusina2.com/en/malattia_ricerca/Database_mutazioni_MFN2 and Inherited Peripheral Neuropathies Mutation Disease (http://www.molgen.ua.ac.be/cmtmutations/Mutations/Mutations.cfm) as well as PubMed. The review by Stuppia was also exploited^44^. Of all 68 identified mutations, those resulting in amino acid in the same position being replaced by at least two other amino acids were selected and used for further analysis (Table 1). Due to the lack of clinical data, p.Arg104Leu and p.Thr105Ala variants^45^ were excluded from our analysis. Moreover, we did not include double mutation in cis (p.Thr105LeufsX2 and p.Phe223Tyr) reported by Park^46^.

### CMT2A symptoms evaluation

To be able to analyze and compare the CMT2A clinical data reported by various groups, which used different examination schemes we developed an internal CMT2A scale based on clinical descriptions of patients found in the literature. Evaluation of neurological symptoms was standardized beginning from basic/classical CMT2A phenotype consisting of distal muscle wasting, weakness and sensory disturbances. As a first criterion of CMT2A severity we used the parameter of age at onset. In general the early age at onset in neurodegenerative disorders serves as a biomarker of clinical severity. Patients manifesting with CMT2A symptoms in early childhood display more severe phenotype than the late onset patients^1,2^.

In addition to the classical symptoms of CMT2A, a clinical variety with uncommon presentation was also included to our internal scale i.e. central nervous system impairment, optic nerve atrophy, and mental retardation, etc. For central nervous system involvement we started from subclinical lesions (MRI abnormalities) and finished on the presence of pyramidal signs or even mental retardation observed in some patients. In fact we cannot definitively exclude that the association between central nervous system involvement and mental retardation is rather casual than causal^11^. Moreover, we include in our scoring scale systemic nature of mitochondrial disorder manifesting with numerous symptoms including myopathy, demyelinating changes in the white matter in the central nervous system and cranial nerve involvement manifested as hearing impairment or visual loss. The others symptoms like vasomotor troubles or cataracts included in our score may not be pathophysiologically associated with CMT2A mitochondrial lesion, but we could not exclude this possibility.

We have used a following internal scoring scale: 1.5 - early onset before age of 5; 1 - age of onset between 6 and 20 years and classical CMT2A without additional symptoms; 0.5 - late age of onset >20 years; 2 - pyramidal signs/extensor plantar responses; 1.5 - mild pyramidal signs; 1.5 - sudden visual loss; 1 - optic nerve atrophy; 1 - mental retardation/developmental delay; 1 - wheelchair-bound; 0.5 - subcortical lesions in MRI; 0.5 - other symptoms i.e. hearing impairment/deafness, sensorineural hearing loss, cerebellar ataxia, vasomotor troubles, tremor, cataracts, learning difficulties, mitochondrial myopathy, microcephaly, periventricular leukomalacia, proximal weakness, markedly reduced nerve conduction velocity in the motor fibers of the median nerve.

### Molecular modelling

Development of a mitofusin 2 structure used for molecular modelling is described in our previous work^11^. Monomeric, GTP-bound BDLP-derived and dimeric models^10^ were refined using to newly available crystallographic data of the MFN2 closest homolog – MFN1^16^. The apo-form of MFN2 was modelled basing on 5GO4 structure^16^. In mechanistic considerations we also included the newly discovered dimeric conformation directly connected to GTP hydrolysis, in which the interface between subunits tightens and HR1 domains are positioned closer to each other. The HR2 position in such conformation remains unknown^20^.

In agreement with the current mechanistic model of mitofusins, structural rearrangements during their activity cycle monomeric forms were modelled in open conformations, while the dimer was modelled as a closed, diamond-shaped complex.

Refined structures were directed to molecular dynamics simulations lasting 10 ns run with careful thermalization and equilibration. All simulations were carried out at 310 K, under atmospheric pressure, and with 0.05 M ionic strength in a rectangular cuboid. The NAMD2 library^47^ with CHARMM27 force field was used^47–50^ and TIP3P water model was applied.

Every considered mutation was introduced into apo-form and dimeric structures or additionally to GTP-bound monomeric state and adjusted transitional states, when needed. At least three runs of each simulation under the parameters described above, were carried out lasting 2 to 10 ns, depending on the obtained results. For each run, an energetic and structural analyses were performed to determine stability of the systems and then mutated region interactions were investigated thoroughly. For mutations placed directly in ligand-binding regions, additional semi-flexible molecular docking of GTP was carried out with MOE 2016.08 software in a AMBER10:EHT force field and directed to molecular dynamics, as described. In order to observe intermolecular interactions driving the close-shutting of the structure, additional 10 ns molecular dynamics simulations of GTP-bound wild-type monomeric MFN2 were performed. In these, the structure was slightly opened by HR2/Paddle rotation at arbitrary selected HR1/HR2 hinges (Gln386-Gln387 and Ala715-Ala716) creating 6 Å gap between GTPase and paddles.

The severity of investigated mutations in computational part of work was assessed basing on the analysis of changes in systems that they triggered. All cases were compared to the reference, which was the WT system in the same particular state. We have taken into consideration the disruption of molecular interactions, formation of new ones and changes in their chemical character and/or energy. We have also analyzed structural changes involving backbone (those are generally more significant for overall structure stability) or sidechains (those are responsible for specific biological functions). All those factors were analyzed including the knowledge of biological mechanisms involving particular structures. When the greater change in the interactions or structure due to the substitution was observed, the higher severity was ascribed. Those comparisons were restricted only to the substitutions placed in the very same residue position.

### Prediction of clinical outcome severity by mutation assessment tools

To predict the severity of analyzed mutations in conventional, commonly used approach the following mutation assessment tools were used: PROVEAN, Fathmm, MutationTaster, PolyPhen-2 and Mutation Assessor. The MutationTaster performs analysis based on nucleotide sequence, the rest of them use the amino acid sequence.

## Electronic supplementary material

Detailed structural analysis of molecular dynamics simulations
