# Supplementary material for: Molecular modelling of mitofusin 2 for a prediction for Charcot-Marie-Tooth 2A clinical severity
Source: Sci Rep. 2018 Nov 15;8:16900. doi: 10.1038/s41598-018-35133-9 (PMC6237821; doi:10.1038/s41598-018-35133-9)
Supplement: Supplementary file 1 — Detailed structural analysis of molecular dynamics simulations [file 41598_2018_35133_MOESM1_ESM.docx]

# Supplementary information for “Molecular modelling of mitofusin 2 for a prediction for Charcot-Marie-Tooth 2A clinical severity” Beręsewicz M., Charzewski Ł., Krzyśko K.A., Kochański A., Zabłocka B.

## Detailed structural analysis of molecular dynamics simulations

**Disturbance in rearrangement of the GTP binding site**

**His277**


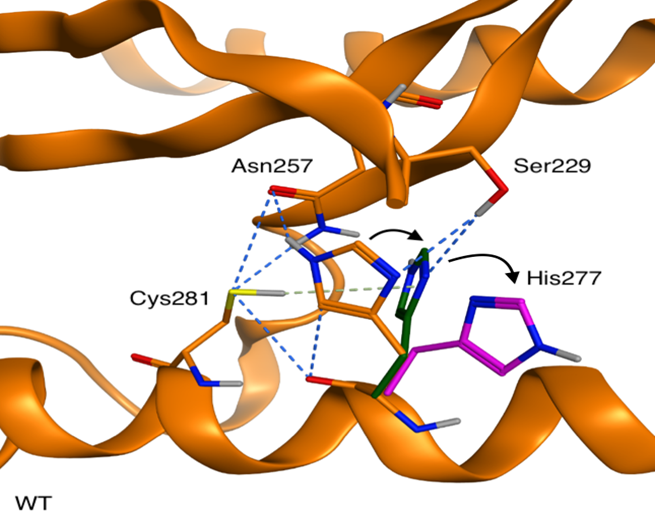


Fig. S1 His277 positioning during different stages of MFN2 activity. In apo form (orange) His277 forms a hydrogen bond (blue dashed line) with Asn257. In GTP-bound monomeric form (green) histidine forms a new hydrogen bond with Ser229 and additional H-pi interaction (fair green dashed line) with Cys281. In dimeric structure (magenta) His277 rotates further, keeping the hydrogen bond with Ser229 but breaking the H-pi interaction with Cys281. Only the apo form secondary structure is presented, for clarity.

In the apo form of wild type, His277 is stabilized by hydrogen bonding with Asn257. During the rearrangement of the GTP binding site, histidine forms a hydrogen bond with Cys281 and Ser229, losing previous interaction with Asn257. While, dimerization leads to a further histidine rotation resulting in keeping only the hydrogen bond with Ser229 side chain (Fig. S1).

In the apo form of mutated MFN2, Arg277 correctly forms a hydrogen bond with Asn257 however, during the rearrangement of GTP binding site and dimerization process, hydrogen bonding is formed with the Ser229 backbone instead of its side chain. In addition, in the dimer we observed the hydrogen bond between Arg277 and Ser229.

The p.His277Tyr mutation forms a hydrogen bond with the Asn257 side chain and a pi-H-type interaction with the Cys281 side chain. In the monomeric system after GTP binding, Tyr277 forms a hydrogen bond with the Ser229 backbone, but in simulations it is formed more often with the backbone Ser231. In the dimeric form, similar to the p.His277Arg mutation, we observed hydrogen bonding with the Ser229 backbone.

In both discussed cases, mutations seem to disrupt the cascade of conformational changes related to the rearrangement of the GTP binding site.

**Impaired dimerization**

New facts on the rotation of MFN1 subunits in its dimeric structure, obtained on the basis of crystallographic studies have been implemented into our previously described model^1,2^. As a result, the interface between paddle regions changed drastically, as they form a contact at different sides of the domains (contacts are formed between regions 644-663 instead of 616-634). The interface shift allowed to conserve interaction located in the center of the GTPase domain, which is crucial for the complex formation (Glu230 - Arg259) and revealed new contacts not present in the previous model (Lys120-Glu266, His165-Glu268, His168-Glu272). Regarding to crystallographic data the apo-form was also modified. The uniqueness of the apo-form is that orientations of Arg259 and Trp260 are swapped, what shelters Arg259 (and Glu266 as well) from the solvent and places Trp260 in a GTP-binding site. Ligand binding is associated with the rearrangement of this particular place.

**Arg259**

In the apo form of the wild type MFN2, the Arg259 and Trp260 side chains are facing in opposite directions from the backbone. The Trp260 side chain is in the GTP binding pocket at the point of attachment of the guanine rings. As a result of GTP entry from the bottom to the binding site, the Trp260 rings are pushed up resulting in rotation of tryptophan and its neighboring Arg259 by approx. 180 degrees. This rotation causes Arg259 takes the place above the GTP rings, creating water protection for them and keeping GTP at the binding site. Arg259 is kept in this position by the so-called salt bridges from the Glu230 of the same subunit and from the Glu230 of the second one.

In the apo form of the p.Arg259Leu mutant, interactions with Glu230 and Glu266 (belonging to the same subunit - see description of the WT for Arg274) are not formed. Interaction with Glu266 is compensated by Arg334, whereas Glu230 is turned away from the protein and ready to interact via one hydrogen bond and electrostatic interaction with the Arg259 of the other subunit (if the wild allele is expressed) . The only hydrophobic partner for Leu259 in the apo form is Ala264 in this place. Additionally, in our simulations we observe that the ligand (the basic part) gains some freedom of movement inside the pocket, which can additionally impede the hydrolysis.

As a result of the mutation p.Arg259Cys, in molecular dynamics simulations we observed the same disturbances of interactions as described for the p.Arg259Leu. Furthermore, we observed the initiation of the rearrangement of the ligand-binding site in the apo form. Cys being a much smaller amino acid than arginine, at position 259 in the apo form cannot reach Glu230 to form a hydrogen bond with it. As a consequence, in this form, we did not observe any interactions of its side chain. This allows its side chain to slide under the protein loop in this position. Simultaneously, the neighboring Trp260 rotates by approx. 90 degrees (both the backbone and side chain) heading towards Glu230, so that a space for a ligand in the GTP-binding pocket appears. This movement probably corresponds with the first stage of pocket rearrangement taking place in physiological conditions. Although the rearrangement is initiated and the protein could begin to carry out the enzymatic activity and dimerization, as the amino acid 259 is critical for dimer formation, the dimerization is hindered and the observed effect is the same as in the p.Arg259Leu mutation.

In biological systems histidine is able to occur in four different protonation states. In the locations considered under physiological conditions its anionic form is highly unlikely to occur, hence only cationic and two neutral protomers have been analyzed. In the p.Arg259His apo form none of these is able to hold Glu230 positioned to the interior of the protein. In the dimeric form histidine most likely occurs as a positively charged residue, partially gaining chemical properties of WT arginine. In our simulations, His259 forms a salt bridge with Glu230 from the other subunit. This interaction is slightly weaker than in the WT and since arginine formed contacts with Glu230 from both subunits, this arrangement might be less stable.

**His165**

As described for the homologue-mitofusin 1, His165 together with His168, belongs to the interface between the subunits in a dimeric form (MFN1: His147-Asp251 and His144-Glu247)^2,3^. However, these interactions are not described as crucial for dimerization. Both histidine residues are in an electrically neutral form, therefore the interaction is not of ionic but of hydrogen bond type (MFN2: His165-Glu268 and His168-Glu272 - Fig. S2).

The p.His165Asp mutation results in an adverse electrostatic interaction with Glu268, which disturbs dimer formation. Furthermore, the interaction His168 - Glu272 is broken, as Glu268, attempting to interact, rotates to Arg275 by intercepting His168 along the way.

As a result of the p.His165Arg mutation, the interaction with Glu268 arises, but it is of a stronger character - it is a hydrogen and an electrostatic interaction simultaneously (the so-called salt bridge). This substitution doesn’t disturb any other interactions.

Substitution of histidine 165 by leucine or tyrosine disables formation of interaction with Glu268, and there are not observed any additional effects of this mutation in simulations.


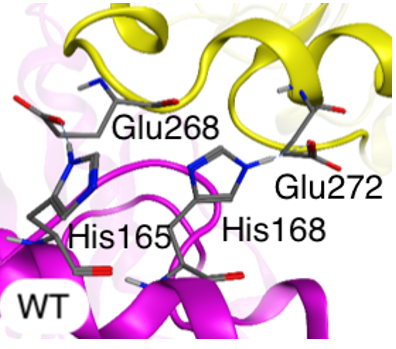


Fig. S2 Intermolecular interactions stabilizing the dimer interface in WT MFN2 (subunits are shown in yellow and pink). His165, His168, Glu268 and Glu272 are shown as sticks, hydrogen bonds are represented with blue dashed line.

**Arg104**

Arg104 is located close to the sites of contact with the second subunit in the dimer however, it is not directly involved in this interaction. Arg104 forms two hydrogen bonds with Glu237, which hold it rigidly at its own subunit. On the other hand, arginine from two MFN2 subunits are located directly against each other. This was shown to be important in the MFN1 structure, that during GTP hydrolysis their structural equivalents interact with each other creating an unfavorable electrostatic effect which might initiate dimer disintegration^1^.

Molecular dynamics revealed that in p.Arg104Trp, the tryptophan side chain, characterized by aromatic properties, moves away from Glu237 and turns towards the other subunit forming stacking interaction.

p.Arg104Gln substitution results in the formation of a similar system as with native arginine - one hydrogen bond is formed with Glu237, thanks to which the amino acid is maintained in a position characteristic for the WT. After interface “tightening” connected with hydrolysis, those residues seem not to influence the dimer stability.

**Arg274**

**
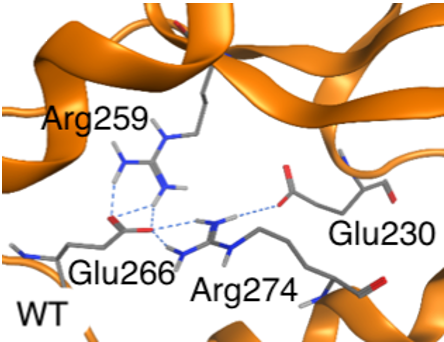
**

Fig. S3 Intermolecular interactions stabilizing MFN2 in the apo form prevent its dimerization.

In the apo form, Arg259 interacts with Glu266 and Glu230, directing them to the interior of the protein - making them inaccessible to the second subunit, hence the dimerization cannot occur (Fig. S3). The GTP entrance to the binding site correlates with the rupture of these interactions (possibly at the same time as the Trp260 rotation occurs), i.e. Arg259 interacts with Glu230 and Arg274 reverts to Glu337 to form a salt bridge interaction. Glu266 form interaction of the same type with Lys128.

The p.Arg274Gln mutation in the apo form does not create interactions with Glu266 and Glu230. One of these two glutamates is held in the interior of the protein by Arg259 (but only one - depending on a simulation, we observe the interaction of one and another alternatively). After ligand binding, Gln274 forms an interaction with Glu337. The sidechain in p.Arg274Trp is not able to generate any of interactions observed in WT.

**Gln276**

As Yan and coworkers described, after MFN1 dimerization, among others, a hydrogen double bond occurs between the Gln255 side chain and the His107 backbone^3^, which corresponds to Gln276 and His128 in MFN2.

The p.Gln276Arg substitution causes the formation of a salt bridge with Glu272. As a result, Glu272 becomes inaccessible to His168 and one of the inter-subunit contacts is lost disturbing the dimerization.

The result of the p.Gln276His mutation is the same as in p.Gln276Arg (i.e., loss of Glu272 - His168 interactions), but the interaction between His276 and Glu272 is weaker - only hydrogen bond.


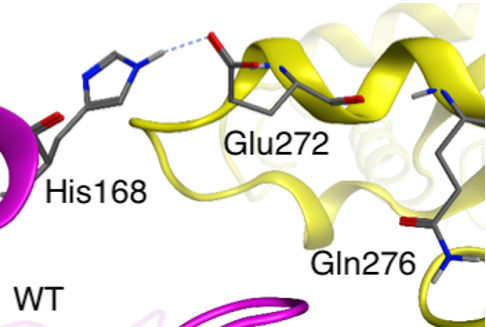


Fig. S4 Intermolecular interactions stabilizing the dimer interface in WT MFN2 (subunits are shown in yellow and pink). Gln276 position in WT MFN2 dimer. The p.Gln276Arg mutation renders Glu272 towards Arg276 displacing it from a proper dimer interface.

**Impaired GTP hydrolysis**

**Gly127**

His128 participates in GTP hydrolysis^1^. In the apo form it is separated from the water by Lys171. At the time of GTP binding, the His128 rotation begins. As a result of dimerization, His128 is rotated further (Fig. S5A), and its correct positioning in the place of operation guarantees precise setting of the water molecule for hydrolysis and suggestions of its involvement in charge compensation during this process were postulated^1^. Mutagenesis of the analogical residue in MFN1 - His107 revealed that its role in GTP hydrolysis is less critical since substitution for alanine decreased GTPase activity in less than 50%^3^. In addition, the water placement is probably still dependent on Gly127, which by analogy to GTP hydrolysis mediated by elongation factor-Tu protein, interacts with the water molecule *via* the nitrogen atom ^4^.

Valine (p.Gly127Val) has the same chemical character as glycine, being only greater in size. Since nitrogen is directed to the GTP-binding site from the backbone (as in WT), this mutation does not disturb the apo form. The obstacle occurs when His128 is rotated, because the hydrophobic interaction of Val127 with Thr105, which arises during rotation and is not present in the WT, increases the rotational energy barrier (Fig. S5B). However, the simulations indicate that if the rotation occurs, the hydrolysis still can be performed because the setting of these atoms does not change as a result of the mutation due to the backbone-only involvement.

Aspartic acid in the apo form (p.Gly127Asp) has a side chain directed outside the GTP- binding cavity (as in WT), which is why GTP binding is not abolished despite such a drastic change in the amino acid chemical properties. However, when dimerization occurs, Asp127 forms an interaction with Gln255 from the other subunit. This interaction causes the displacement of the loop on which His128 is located, therefore GTP hydrolysis is impaired (Fig. S5C). In addition, the above interaction destroys the His168 - Glu272 interaction identified as participating in a dimer formation.

**
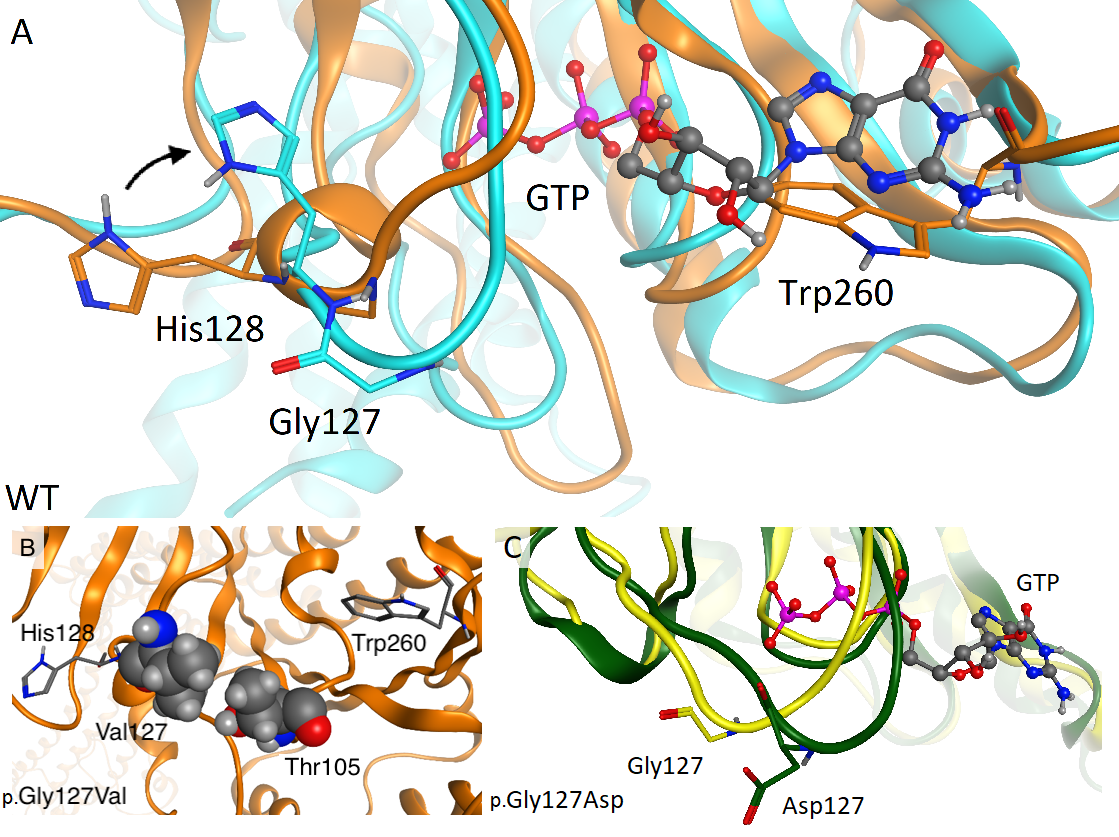
**

**Fig. S5** Gly127 mutations. **A)** Gly127 and His128 positions in apo (orange) and dimeric GTP-bound (blue) form of MFN2. GTP molecule is represented with balls and sticks and amino acids with sticks. For clarity, Trp260 position in the apo form is shown. **B)** In a mutated apo form of MFN2 Val127 forms Van der Waals interaction with Thr105 (shown as VdW spheres). Additionally, His128 and Thr260 are depicted. **C)** Structure of dimeric mitofusin carrying p.Gly127Asp mutation (green). Mutation causing visible loop displacement in comparison with wild type (yellow).

**Impaired MFN2 closure**

**Asp210**

In the p.Asp210Val mutation, one electrostatic interaction between GTPase and HR2 domain (Arg476) is lost. Thus, the structure closes with a greater difficulty or might not close at all. Tyrosine is a much larger amino acid than aspartic acid, therefore as a result of p.Asp210Tyr mutation MFN2 loses not only the salt-bridge with Arg476, but also the Lys243 - Asp480 interaction (paddle tightening) because Tyr210 causes a steric hindrance. As a result the structure closes with a greater difficulty and the “tightening” mechanism is weaker in comparison with the p.Asp210Val mutation.

**Arg250**

The molecular effect of the p.Arg250Gln mutation is the absence of one electrostatic interaction between GTPase and HR2 domain (with Glu598). Although a hydrogen bond interaction may form, it is much weaker and of a smaller range what may result in difficulties in structure closure.

Tryptophan (p.Arg250Trp) is a much larger residue than arginine. In the immediate vicinity of this interdomain interaction, there are only hydrogen bonds “tightening” HR2 and thus can be destroyed as a result of a steric obstacle. Structure analysis revealed that tryptophan substitution may have greater effect on the interdomain interaction than glutamine.

Table S1 Comparison of clinical outcome, structure impairment (due to mutations position and properties of substituted amino acids) and predicted clinical outcome (based on mutation assessment tools).

| **Amino acid position** | **Clinical outcome** | **Structure impairment** | **PolyPhen-2**  **HumDiv*^a^*** | **PolyPhen-2**  **HumVar*^b^*** | **Mutation Assessor*^c^*** | **PROVEAN*^d^*** | **Fathmm*^e^*** | **MutationTaster*^f^*** |
| --- | --- | --- | --- | --- | --- | --- | --- | --- |
| **Arg104** | Trp>Gln | Trp>Gln | Trp=Gln | Trp>Gln | Trp>Gln | Trp>Gln | Trp>Gln | Trp=Gln |
| **Gly127** | Asp>Val | Asp>Val | Asp=Val | Asp=Val | Asp>Val | Val>Asp | Asp=Val | Asp=Val |
| **His165** | Asp>Arg>Leu=Tyr | Asp>Arg>Leu=Tyr | Asp=Arg=Leu=Tyr | Asp=Arg=Leu=Tyr | Tyr>Leu>Arg>Asp | Leu>Asp>Arg>Tyr | Asp=Arg=Leu=Tyr | Asp=Arg=Leu=Tyr |
| **Asp210** | Tyr=Val | Tyr>Val | Tyr>Val | Tyr>Val | Tyr>Val | Tyr=Val | Tyr=Val | Tyr=Val |
| **Val244** | Leu$\geq$Met | Leu=Met | Met>Leu | Met>Leu | Met>Leu | Leu=Met | Met>Leu | Leu=Met |
| **Arg250** | Trp>Gln | Trp>Gln | Trp>Gln | Trp>Gln | Trp>Gln | Trp>Gln | Trp=Gln | Trp=Gln |
| **Pro251** | Arg>Ala>Leu | Arg>Ala=Leu | Arg=Ala=Leu | Arg=Ala=Leu | Leu=Ala>Arg | Leu>Arg>Ala | Ala=Leu>Arg | Arg=Ala=Leu |
| **Arg259** | Leu$\geq$Cys>His | Leu=Cys>His | Leu=Cys=His | Leu=His>Cys | Leu=Cys=His | Cys>Leu>His | Leu=Cys=His | Leu=Cys=His |
| **Arg274** | Trp>Gln | Trp>Gln | Trp>Gln | Trp>Gln | Trp>Gln | Trp>Gln | Trp>Gln | Trp=Gln |
| **Gln276** | Arg=His | Arg=His | His>Arg | His>Arg | Arg>His | His>Arg | Arg=His | Arg=His |
| **His277** | Tyr>Arg | Tyr=Arg | Arg>Tyr | Arg>Tyr | Tyr>Arg | Arg>Tyr | Tyr>Arg | Tyr=Arg |
| Compatibility of clinical outcome and predicted clinical outcome | | 73% | 18% | 27% | 45% | 45% | 45% | 27% |

*^a, b^* **PolyPhen-2** classifies substitutions, as ”benign”, ”possibly damaging” or ”probably damaging” based on pairs of false positive rate thresholds. *^a^* **HumDiv** is preferred model for evaluating rare alleles, dense mapping of regions identified by genome-wide association studies, and analysis of natural selection. *^b^* **HumVar** is preferred model for diagnostics of Mendelian diseases which requires distinguishing mutations with drastic effects from all the remaining human variation, including abundant mildly deleterious alleles.

*^c^* **Mutation Assessor** classifies substitutions as high functional (score 3.50-5.50), medium functional (score 2.00-3.50), low non-functional (score 1.00-2.00) or neutral non-functional (score below 1.00)

*^d^* **PROVEAN** (Protein Variation Effect Analyzer) v1.1 classifies substitutions as deleterious (score is equal to or below a predefined threshold -2.5) or neutral (score is above the threshold)

*^e^* **Fathmm** classifies substitutions as ”damaging”(score below predefined threshold -1.5) and ”tolerated” (score above threshold)

*^f^* **MutationTaster** classifies substitutions as ”disease causing/ probably deleterious”, ”disease causing automatic/ known to be deleterious”, ”polymorphism/ probably harmless”, ”polymorphism automatic/ known to be harmless”

Reference List

1 Cao, Y. L. *et al.* MFN1 structures reveal nucleotide-triggered dimerization critical for mitochondrial fusion. *Nature* **542**, 372-376, doi:10.1038/nature21077 (2017).

2 Beresewicz, M. *et al.* The Effect of a Novel c.820C>T (Arg274Trp) Mutation in the Mitofusin 2 Gene on Fibroblast Metabolism and Clinical Manifestation in a Patient. *PLoS.ONE.* **12**, e0169999 (2017).

3 Yan, L. *et al.* Structural basis for GTP hydrolysis and conformational change of MFN1 in mediating membrane fusion. *Nat Struct Mol Biol* **25**, 233-243, doi:10.1038/s41594-018-0034-8 (2018).

4 Voorhees, R. M., Schmeing, T. M., Kelley, A. C. & Ramakrishnan, V. The mechanism for activation of GTP hydrolysis on the ribosome. *Science* **330**, 835-838, doi:10.1126/science.1194460 (2010).
